# The growth of education differentials in marital dissolution in the United States

**DOI:** 10.4054/demres.2021.45.26

**Published:** 2021

**Authors:** Kim McErlean

**Affiliations:** 1 University of Texas at Austin, USA

## Abstract

**BACKGROUND:**

Recent data suggest that overall divorce rates in the United States have been declining since the 1980s, while research examining marriages formed prior to 2004 suggests that divorce rates historically have not declined equally across the socioeconomic spectrum. Understanding recent differentials by education helps explore growing inequality over time given the well-documented negative consequences of divorce for women.

**OBJECTIVE:**

This study examines marital dissolution and divorce rates in the new millennium to understand trends by marital cohort and educational attainment.

**METHODS:**

To understand recent trends in marital stability, this study uses the 2006–2019 National Survey of Family Growth female dataset to assess the likelihood of marital dissolution and divorce by the 5^th^ and 10^th^ anniversary. Life tables and discrete-time event-history analyses are used to measure marital dissolution over time and by educational attainment while controlling for risk factors that may explain differentials.

**RESULTS:**

Overall marital dissolution and divorce rates are declining over time. However, this downward trend is driven by those with higher education; those with the least education are seeing rising marital dissolution rates, even when controlling for correlated risk factors. The greater divide when examining marital dissolution as compared to formal divorce also illustrates the lower propensity of the least educated to formalize their dissolution.

**CONTRIBUTION:**

Overall dissolution trends hide important – and growing – differentials by educational attainment. Declines in dissolution are not equally distributed across social classes; those women who are most vulnerable to divorce are least likely to be able to recover from its negative consequences.

## 1. Introduction

Divorce is a powerful contributor to gender and class stratification. Women lose more financially in divorce than men (Sayer 2005; Tach and Eads 2015), and women with the least resources face the highest risk, reinforcing a cycle of inequality (Amato 2000; McLanahan and Percheski 2008). Despite a popular perception that half of American marriages end in divorce (Cohen 2017; Luscombe 2018), rates have been declining since the 1980s (Cohen 2019; Kennedy and Ruggles 2014). Overall declines in divorce allow for cautious optimism around the state of marriages today, if all social classes are experiencing declines. Overall trends might be masking important class differentials such that women with the least education, who are less financially prepared for the consequences of divorce, are experiencing rising dissolution rates.

Historical declines in divorce did not occur for all. Martin (2006) uses the Survey of Income and Program Participation (SIPP) to show that marital dissolution rates declined for marriages formed between 1975 and 1994 overall and for those with some college experience but rose 8% for those without a high school diploma. Raley and Bumpass (2003) show similar trends using the National Survey of Family Growth (NSFG): Dissolution rates declined for college graduates who married between 1980 and 1994 but rose 5% for those without high school degrees. Schwartz and Han (2014), using the NSFG and Panel Study of Income Dynamics (PSID), confirm that this divergence extended to marriages formed through at least 2004. Looking abroad, Härkönen and Dronkers (2006) find that divorce was declining more rapidly for the most educated in 9 out of 17 countries.

I am unaware of research examining educational differentials in the United States since the early 2000s, so it’s unclear if this gap continues to grow. There is reason to think that it may have shrunk. Marriage rates have declined overall, but particularly among economically disadvantaged women (Manning, Brown, and Payne 2014), causing some researchers to suggest that couples marry only when they feel certain it will last (Smock, Manning, and Porter 2005), thus potentially leading to declines in marital dissolution among the least educated. Yet as income inequality and economic precarity for low-class families has grown, this gap may persist (Cooper and Pugh 2020).

Another question is whether total marital dissolution rates (including divorce and separation) have declined with divorce rates; less research has examined patterns over time for both outcomes. This is particularly important when considering class differences, as those with less education and fewer resources are less likely to formally divorce, likely driven by costs (Bennett 2017; Tumin and Qian 2017). Sweeney and Phillips (2004) find that Black women had 5%–10% higher divorce rates than White women, but nearly 25% higher rates of total marital dissolution. Lack of formal divorce inhibits remarriage, the most common way that women recover financially after divorce (Morrison and Ritualo 2000); understanding how divorce and separation vary by educational attainment furthers our understanding of the reproduction of inequality.

This study adds recent evidence on education differentials in marital stability in the United States by comparing trends in marital dissolution and divorce by educational attainment for marriages formed between 2000 and 2014.

## 2. Data and methods

### 2.1 Data

I use the National Survey of Family Growth (NSFG), cycles 2006–2019. The NSFG is a nationally representative, cross-sectional survey of women aged 15 to 44 (49 after 2015) that asks detailed questions on relationship and fertility history. I use only the female dataset as marital event histories tend to be more accurate for women (Kennedy and Ruggles 2014); only women who have been married are included. I focus on first marriages as the consequences of first divorce are most established; all marriages are heterosexual as the NSFG does not collect data on same-sex marriages. Following others using the NSFG (e.g., Bramlett and Mosher 2002; Kuperberg 2014), I remove respondents who married more than 10 years before the interview or after the age of 35. These restrictions help mitigate age at marriage biases that could arise because of the NSFG’s retrospective nature coupled with its age limit of 15 to 44. To be captured in later cycles of the NSFG, those who married in earlier cohorts had to have married at younger ages, which is a risk factor for dissolution. These restrictions have been questioned with regard to their implications on generalizability (see Manning, Smock, and Kuperberg 2021; Rosenfeld and Roesler 2021). However, I find that these restrictions still leave 75% of the eligible sample and that results were substantively similar without restrictions, so I continue with the restricted sample as this bias is not evenly distributed across my sample; the less educated are more likely to marry at younger ages. Finally, I remove cases with missing values on key variables; these cases make up less than 0.5% of my sample, leaving an analytical sample of 6,356 women.

The primary dependent variable is marital dissolution, both separation and divorce; I compare to divorce in the descriptive analysis to understand differences between the two outcomes. Respondents who were censored or not dissolved at the time of the survey are coded as 0; those who dissolved are coded as 1. To understand trends since 2000, the key independent variable is five-year marital cohort: 2000–2004, 2005–2009, and 2010–2014. Education describes attainment at the time of interview and has four categories: less than a high school degree, high school degree, some college, and college degree or more.

Although risk of marital dissolution tends to decline by marital duration, the association is nonlinear (Jalovaara and Kulu 2018; Teachman 2011); all models include a discrete measure of duration to allow complete flexibility in the duration function. Multivariate analyses also control for characteristics that have historically been associated with marital dissolution. These include age at marriage and its square, based on an initial analysis using discrete age; premarital cohabitation; premarital fertility; region of residence; and race/ethnicity.

### 2.2 Analytical approach

I first assess trends over time, describing the Kaplan-Meier estimate of the proportion of respondents that dissolved their first marriage within 5 years (all cohorts) and 10 years (the first two cohorts). Analyses compare marital dissolution and divorce across marital cohorts by educational attainment. I then use discrete-time event-history analysis to assess these trends net of covariates. I convert my data into person-years, yielding 28,356 person-years of analysis. The outcome variable is a binary indicator of whether the respondent dissolved their marriage in each year up until their fifth anniversary. I use the fifth anniversary for the event-history analysis so I can include the 2010–2014 cohort and compare trends to prior research that used this dataset and methodology (Raley and Bumpass 2003). As life table estimates in Section 3 show, trends in education differentials are similar using 5- or 10-year estimates: Both show that differentials have widened since 2000–2004. I take a discrete-time approach because marital history data is available at only a yearly level in the most recent public-use NSFG datasets. Following Allison’s (1982) recommendation for processes that happen continuously, but are only measured discretely, I estimate logit models as well as complementary log-log models; results are nearly identical, so I present results from the logit models. All analyses are weighted using NSFG sample weights; each cycle’s weights are rescaled to a mean of 1 before combining to give the appropriate weight to each cycle based on its sample size (Heeringa, West, and Berglund 2017).

## 3. Results

### 3.1 Divorce and marital dissolution rates since 2000

Table 1 shows life table estimates of marital dissolution and divorce by marital cohort. Prior estimates using the NSFG show marital dissolution risk by fifth anniversary was 22% in 1994 (Raley and Bumpass 2003). These more recent estimates illustrate that marital dissolution rates have declined further in the new millenium, with an especially large decline for the latest cohort. The risk of dissolution at five years rises slightly for those who married during the recession, but the decline resumed afterward. The recession is likely an outlier in terms of instability. Past research finds that couples who experience economic instability in the early years of marriage have a higher risk of divorce (Preston and McDonald 1979). Those who married during the recession may have been more likely to dissolve as a result of early economic strains. Divorce rates follow a similar trajectory with lower incidence as not all couples divorce upon separation. These data support findings that divorce rates have been declining over time and add to prior research by showing that marital dissolution follows the same pattern.

### 3.2 Divorce and dissolution rates by educational attainment

Life table estimates in Table 2 show that declining marital dissolution rates are not occuring equally across groups. Dissolution rates are declining only for college graduates. The least educated women see a rise in dissolution over time, exacerbating the divide in marital stability. The 12% difference in 5-year marital dissolution rates between the most and least educated in 2000–2004 grew to 23% by 2014; 10-year risk diverged even more between 2000–2004 and 2005–2009.

Turning to differences between marital dissolution and divorce (Table 3), differentials for divorce are smaller than for marital dissolution, though the gap is growing for both outcomes. The gap in five-year divorce rates in 2010–2014 is only 11%, compared to 23% for total marital dissolution, suggesting that the least educated are more likely to informally separate rather than divorce. This highlights the point that analyses that measure only formal divorce are likely overstating marital stability but underestimating educational differentials.

Perhaps surprisingly, in some cohorts, those with high school degrees and some college have higher divorce rates than those with less than high school degrees. This aligns with other research focusing on divorce specifically (see Cohen 2019) and serves to reinforce the point that the least educated are particularly unlikely to formalize their dissolution with a divorce, but their marriages are more unstable on the whole.

I use discrete-time event-history analysis to confirm if this widening of rates persists when controlling for factors historically associated with dissolution. Table 4 presents odds ratios from a series of logistic regressions predicting marital dissolution. Model 1 includes marital duration and cohort to capture overall declines in dissolution. The odds ratio for the 2010–2014 cohort is lower than that for 2000–2004; calculating predicted marginal effects, holding all other variables at their means, women who married in 2010–2014 have 30% lower relative probability of dissolution than those who married in 2000–2004. Model 2 adds education to describe educational differences. Women with less than a high school degree have higher odds of marital dissolution than those with a college degree. Model 3 adds controls to this model to see if these differences persist, which they do.

**Figure 1:**
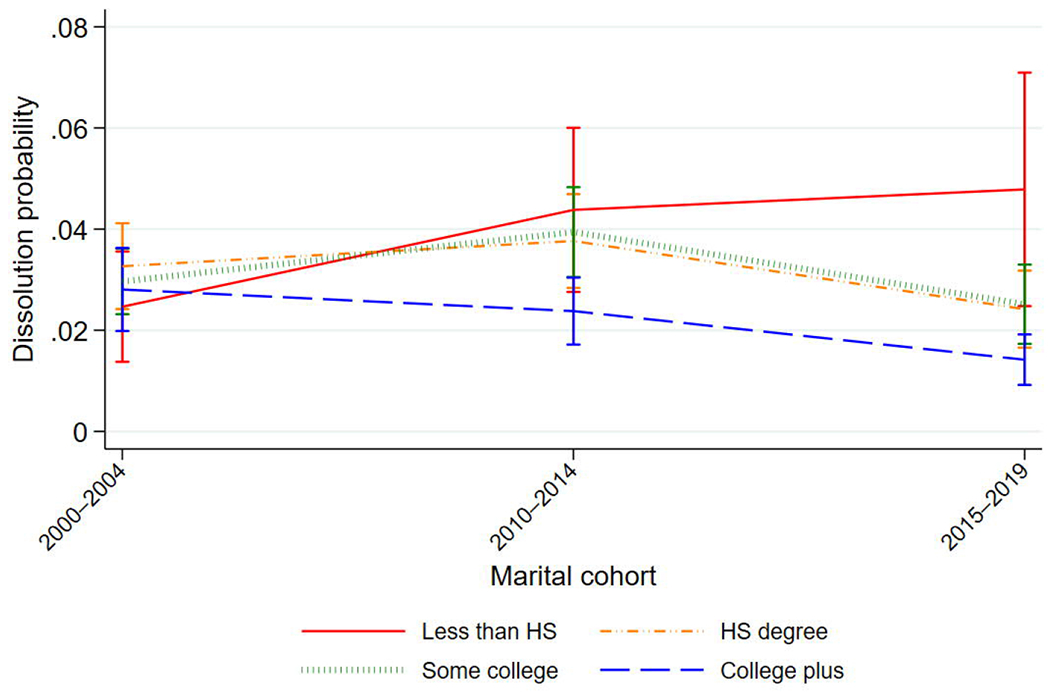
Predicted probability of dissolution by education and marital cohort *Notes:* Figure shows marginal effects (at means) and 95% confidence intervals from the event-history analysis, Model 5.

Model 4 interacts education and cohort to understand if education differentials have changed over time and indicates that they have. The main effect for college graduates indicates that there are education differentials for the 2000–2004 cohort. These estimates are consistent with, though slightly more conservative than, prior research (Schwartz and Han 2014); differences in estimates are potentially driven by their inclusion of remarriages, while I focus on first marriages only, as well as different covariates considered. The negative interaction effect for college graduates and the 2010–2014 cohort demonstrates that these differences have widened over time. Finally, Model 5 adds in all control variables to see if they explain this widening gap. Controls did eliminate the education differentials for the 2000–2004 cohort, but not for those married in 2005–2014, where differences grew with each cohort. By the 2010–2014 cohort, the college educated had a 70% lower dissolution risk than those without a high school degree, based on average marginal effects at the mean. These results confirm that the gap in marital dissolution between the most and least educated has widened over time, and that this gap in recent cohorts cannot be explained by how the measured risk factors vary by educational attainment. Figure 1 shows the predicted probability of marital dissolution using Model 5 to illustrate this widening gap.

## 4. Discussion

This study uses the NSFG to understand the growth of education differentials in divorce in light of overall declining divorce rates in the United States. Results support prior findings that divorce rates are declining and confirm that marital dissolution rates are as well but that overall trends mask important class differences. Dissolution rates have diverged over time between the most and least educated, such that those married in 2010–2014 have the widest gap. Event-history analysis indicates that this divergence could not be fully explained by risk factors included here. Further, the less educated are less likely to formalize their separation with legal divorce. While divorce may be costly, it is necessary for remarriage, which is the surest route to economic recovery for divorced women (Morrison and Ritualo 2000), as well as asset division and child support. Women with the least resources struggle to attain marital stability but are less prepared for the negative financial consequences of dissolution.

These results are potentially surprising, given that marriage rates have been declining for the least educated (Hendi 2019; Manning, Smock, and Payne 2014). Some scholars suggest that as fewer people marry, it has become more of an out-of-reach status symbol for some (Cherlin 2004). If only those who feel financially and emotionally secure marry, we would expect a convergence in divorce rates by educational attainment, opposite to the results here. Future research could look into marriage patterns by class to understand why this divergence in dissolution persists despite declining marriage rates.

This analysis highlights education differentials in marital dissolution that future research could explore to identify potential explanations. First, the analyses demonstrated that the control variables used reduced the differences in education coefficients in 2000–2004, but this was not the case in later cohorts. As such, the relationship between education and dissolution may be no longer be driven by differences in behaviors and characteristics but rather broader structural disadvantages I am unable to measure with the NSFG. More women have attained college degrees as this credential has become more necessary for steady employment. As such, those without even a high school degree might be an increasingly disadvantaged group (Zheng 2020), with even higher risk of dissolution. Research has shown that, as the more educated have more to lose in a divorce, they have become less permissive toward divorce, whereas the least educated, who face day-to-day uncertainty, are more permissive in the face of that uncertainty (Martin and Parashar 2006). This uncertainty has only increased as economic precarity and income inequality have been growing (Thompson and Smeeding 2013). Finally, as marriage rates have been declining, marriages may have become concentrated among those who hold traditional beliefs about marriage. These beliefs usually correspond to younger ages at marriage (Uecker and Stokes 2008) and correlate with other indicators of structural disadvantage (Glass and Levchak 2014), which may outweigh the potential protective effect of these beliefs, such that these marriages might be particularly unstable.

Some limitations of the NSFG prevented me from fully explaining these differentials. I used education as a proxy for socioeconomic status; the NSFG does not have comprehensive measures of socioeconomic status, such as employment or income. The NSFG does not include measures of relationship quality or behaviors. Past research has shown that the least educated couples have more severe relationship problems, including higher rates of substance abuse and intimate partner violence that may hamper relationship stability (Trail and Karney 2012). As noted, I restricted my sample such that marriages of longer durations and those who married beyond the age of 35 are not captured here, so these trends are not representative of all marriages; these factors likely enhance marital stability. In a similar vein, the NSFG lacks respondents older than 44 (49 after 2015), which restricts me from capturing the role of ‘gray divorce’ in these trends, potentially biasing my estimates downward.

Overall, this study provides new evidence that marital dissolution rates continue to diverge by educational attainment for marriages formed since 2000. These results also highlight the importance of considering both marital dissolution and divorce rates when looking at trends in marital stability; the gap is much wider when considering marital dissolution. The less educated are in a more disadvantaged social position, and marital dissolution often has negative consequences for current and future generations; these results suggest one way that gender and class inequality may be reproduced via family structure.

## Figures and Tables

**Table 1:** Marital dissolution and divorce by marital cohort

	5-year risk			10-year risk		

	2000–2004	2005–2009	2010–2014	2000–2004	2005–2009	2010–2014
Total marital dissolution	14.8%	16.1%	11.0%	25.8%	25.1%	n/a
Formal divorce only	9.7%	11.0%	7.2%	20.4%	20.6%	n/a

*Notes:* Values come from Kaplan-Meier survival function. All values are based on weighted data.

**Table 2:** Risk of marital dissolution over time by education

	5-year risk			10-year risk			Sample

	2000–2004	2005–2009	2010–2014	2000–2004	2005–2009	2010–2014	% of Total
Less than HS	22.0%	26.8%	29.4%	29.0%	41.6%	n/a	11.3%
HS degree	24.8%	23.6%	23.8%	36.4%	29.4%	n/a	23.7%
Some college	20.3%	26.3%	17.1%	28.2%	39.4%	n/a	30.1%
College degree	10.5%	9.4%	5.9%	17.1%	14.2%	n/a	34.9%

*Point difference: less than HS and college degree*	*11.5%*	*17.4%*	*23.5%*	*11.9%*	*27.4%*	*n/a*	

*Notes:* Values come from Kaplan-Meier survival function. All values are based on weighted data. *N* (individuals) = 6,356; *N* (dissolutions) = 1,536.

**Table 3:** Risk of divorce over time by education

	5-year risk			10-year risk		

	2000–2004	2005–2009	2010–2014	2000–2004	2005–2009	2010–2014
Less than HS	10.5%	15.9%	15.8%	21.7%	23.5%	n/a
HS degree	15.9%	22.9%	14.0%	28.6%	28.1%	n/a
Some college	14.9%	18.5%	12.0%	26.6%	28.1%	n/a
College degree	9.2%	7.4%	4.8%	16.8%	12.0%	n/a

*Point difference: less than HS and college degree*	*1.3%*	*8.5%*	*11.0%*	*8.5%*	*11.5%*	*n/a*

*Notes:* Values come from Kaplan-Meier survival function. All values are based on weighted data. *N* (individuals) = 6,356; *N* (divorces) = 997.

**Table 4:** Discrete-time event-history results for marital dissolution (within first five years of marriage)

	Model 1			Model 2			Model 3			Model 4			Model 5		

Variables (reference)	B	OR	95% CI	B	OR	95% CI	B	OR	95% CI	B	OR	95% CI	B	OR	95% CI
Marital duration (1 year)															
2 years	0.32	1.38	1.08–1.76	0.34	1.41	1.11–1.80	0.35	1.42	1.11–1.82	0.35	1.41	1.11–1.80	0.35	1.42	1.11–1.82
3 years	0.15	1.16	0.92–1.45	0.18	1.20	0.96–1.51	0.20	1.23	0.97–1.55	0.19	1.21	0.96–1.52	0.21	1.23	0.98–1.55
4 years	0.14	1.15	0.90–1.47	0.19	1.21	0.95–1.55	0.23	1.26	0.98–1.61	0.20	1.22	0.95–1.55	0.24	1.27	0.99–1.62
5 years	0.16	1.17	0.88–1.55	0.21	1.24	0.93–1.64	0.26	1.30	0.98–1.73	0.22	1.24	0.94–1.65	0.27	1.31	0.98–1.74
Marital cohort (2000–2004)															
2005–2009	0.08	1.09	0.90–1.31	0.14	1.15	0.96–1.39	0.18	1.20	0.99–1.45	0.38	1.46	0.86–2.49	0.59	1.81	1.05–3.11
2010–2014	−0.37	0.69	0.55–0.87	−0.29	0.75	0.60–0.94	−0.23	0.79	0.63–1.00	0.50	1.64	0.89–3.05	0.69	1.99	1.05–3.78
Educational attainment (less than HS)															
HS degree				−0.12	0.89	0.68–1.18	−0.05	0.95	0.71–1.27	0.15	1.16	0.73–1.85	0.30	1.35	0.82–2.20
Some college				−0.27	0.76	0.58–1.00	−0.06	0.94	0.70–1.28	−0.14	0.87	0.55–1.36	0.19	1.21	0.74–1.98
College degree				−1.23	0.29	0.22–0.40	−0.43	0.65	0.44–0.95	−0.76	0.47	0.28–0.77	0.13	1.14	0.65–2.01
Interactions (2000–2004 × Less than HS)															
2005–2009 × Less than HS										–	–	–	–	–	–
2005–2009 × HS degree										−0.30	0.74	0.40–1.39	−0.45	0.64	0.34–1.21
2005–2009 × Some college										−0.08	0.92	0.50–1.70	−0.30	0.74	0.40–1.38
2005–2009 × College degree										−0.56	0.57	0.29–1.11	−0.76	0.47	0.24–0.92
2010–2014 × Less than HS										–	–	–	–	–	–
2010–2014 × HS degree										−0.85	0.43	0.21–0.89	−1.00	0.37	0.17–0.78
2010–2014 × Some college										−0.69	0.50	0.25–1.03	−0.86	0.42	0.20–0.88
2010–2014 × College degree										−1.27	0.28	0.13–0.61	−1.38	0.25	0.11–0.55
Age at marriage															
Age married							−0.44	0.64	0.53–0.78				−0.46	0.63	0.52–0.76
Age married (squared)							0.01	1.01	1.00–1.01				0.01	1.01	1.00–1.01
Relationship behaviors															
Cohabited with husband (did not)							−0.02	0.98	0.81–1.20				−0.01	0.99	0.82–1.21
Cohabited with other (did not)							−0.06	0.94	0.75–1.18				−0.06	0.94	0.75–1.18
Had premarital birth (did not)							0.81	2.25	1.84–2.75				0.81	2.25	1.84–2.76
Race / Ethnicity (White)															
Black							0.74	2.09	1.67–2.62				0.73	2.08	1.66–2.61
Hispanic							−0.31	0.74	0.58–0.94				−0.33	0.72	0.56–0.92
Other							−0.91	0.83	0.55–1.24				−0.18	0.84	0.56–1.25
Urbanicity of residence (Urban)															
Suburb							−0.15	0.86	0.71–1.04				−0.15	0.86	0.71–1.04
Rural							−0.02	0.98	0.76–1.25				−0.02	0.98	0.76–1.25
Constant	−3.37	0.03	0.03–0.04	−2.94	0.05	0.04–0.07	2.89	18.01	1.93–168.13	−3.16	0.04	0.03–0.06	2.84	17.14	1.84–160.08
Person-years	28,356			28,356			28,356			28,356			28,356		
χ^2^				107.99						12.33			233.77		

*Notes:* χ^2^ shows results from the goodness-of-fit tests for each subsequent model. 95% confidence interval is based on odds ratio. All values are weighted.
